# Difference in LET‐based biological doses between IMPT optimization techniques: Robust and PTV‐based optimizations

**DOI:** 10.1002/acm2.12844

**Published:** 2020-03-09

**Authors:** Shusuke Hirayama, Taeko Matsuura, Koichi Yasuda, Seishin Takao, Takaaki Fujii, Naoki Miyamoto, Kikuo Umegaki, Shinichi Shimizu

**Affiliations:** ^1^ Research and Development Group Center for Technology Innovation‐Energy Hitachi Ltd Hitachi‐shi Ibaraki‐ken Japan; ^2^ Graduate School of Biomedical Science and Engineering Hokkaido University Sapporo Hokkaido Japan; ^3^ Division of Quantum Science and Engineering Faculty of Engineering Hokkaido University Sapporo Hokkaido Japan; ^4^ Global Station for Quantum Medical Science and Engineering Global Institution for Collaborative Research and Education (GI‐CoRE) Hokkaido University Sapporo Hokkaido Japan; ^5^ Department of Medical Physics Hokkaido University Hospital Sapporo Hokkaido Japan; ^6^ Department of Radiation Oncology Hokkaido University Hospital Sapporo Hokkaido Japan; ^7^ Department of Radiation Medical Science and Engineering Faculty of Medicine Hokkaido University Sapporo Hokkaido Japan

**Keywords:** plan comparison, proton therapy, robust optimization, variable RBE

## Abstract

**Purpose:**

While a large amount of experimental data suggest that the proton relative biological effectiveness (RBE) varies with both physical and biological parameters, current commercial treatment planning systems (TPS) use the constant RBE instead of variable RBE models, neglecting the dependence of RBE on the linear energy transfer (LET). To conduct as accurate a clinical evaluation as possible in this circumstance, it is desirable that the dosimetric parameters derived by TPS ($D^{\text{RBE}=1.1}$) are close to the “true” values derived with the variable RBE models ($D^{v\text{RBE}}$). As such, in this study, the closeness of $D^{\text{RBE}=1.1}$ to $D^{v\text{RBE}}$ was compared between planning target volume (PTV)‐based and robust plans.

**Methods:**

Intensity‐modulated proton therapy (IMPT) treatment plans for two Radiation Therapy Oncology Group (RTOG) phantom cases and four nasopharyngeal cases were created using the PTV‐based and robust optimizations, under the assumption of a constant RBE of 1.1. First, the physical dose and dose‐averaged LET (LET_d_) distributions were obtained using the analytical calculation method, based on the pencil beam algorithm. Next, $D^{v\text{RBE}}$ was calculated using three different RBE models. The deviation of $D^{v\text{RBE}}$ from $D^{\text{RBE}=1.1}$ was evaluated with *D*_99_ and *D*_max_, which have been used as the evaluation indices for clinical target volume (CTV) and organs at risk (OARs), respectively. The influence of the distance between the OAR and CTV on the results was also investigated. As a measure of distance, the closest distance and the overlapped volume histogram were used for the RTOG phantom and nasopharyngeal cases, respectively.

**Results:**

As for the OAR, the deviations of $D_{\max}^{v\text{RBE}}$ from $D_{\max}^{\text{RBE}=1.1}$ were always smaller in robust plans than in PTV‐based plans in all RBE models. The deviation would tend to increase as the OAR was located closer to the CTV in both optimization techniques. As for the CTV, the deviations of $D_{99}^{v\text{RBE}}$ from $D_{99}^{\text{RBE}=1.1}$ were comparable between the two optimization techniques, regardless of the distance between the CTV and the OAR.

**Conclusion:**

Robust optimization was found to be more favorable than PTV‐based optimization in that the results presented by TPS were closer to the “true” values and that the clinical evaluation based on TPS was more reliable.

## 1. INTRODUCTION

Most newly built proton therapy centers worldwide are implementing the pencil beam scanning technique because of its distinct advantages of dose conformity to targets and neutron exposure reduction compared to the more conventional passive scattering methods. In particular, intensity‐modulated proton therapy (IMPT) enables the creation of highly conformal dose distributions in tumors while sparing nearby organs at risk (OARs) by optimizing the spot intensities from all beams simultaneously.^1^ The optimization techniques of IMPT are categorized into two: planning target volume (PTV)‐based optimization and robust optimization. In robust optimization, the dose distributions for multiple uncertainty scenarios (e.g., setup and range uncertainties) are calculated, and treatment plans are optimized simultaneously with respect to all the scenarios.^2^, ^3^, ^4^, ^5^, ^6^ Both techniques are implemented in commercial treatment planning systems (TPS) and have been used in clinical practice.

To take full advantage of IMPT, it is necessary to incorporate the biological effects of protons in the treatment planning process. In current clinical practice, a proton beam is delivered assuming a constant relative biological effectiveness (RBE) of 1.1. On the contrary, extensive preclinical evidence shows that the RBE varies across treatment fields. Particularly, it depends on linear energy transfer (LET), tissue‐specific parameters (*α* and *β*), dose per fraction, and other factors.^7^ Various phenomenological RBE models considering LET have been proposed,^8^, ^9^, ^10^, ^11^ and these are herein referred to as “variable RBE models.” Some researchers use the variable RBE‐weighted dose in both the calculation and optimization of IMPT,^12^ while others use both the physical dose and the biological surrogate (which is defined as the sum of LET × physical dose and physical dose, yielding values similar to the variable RBE‐weighted dose)^13^ simultaneously in the optimization, to increase LET in tumors.^14^ Furthermore, the biological surrogate is used to avoid the occurrence of high LET areas in critical organs.^15^ However, as far as the authors’ knowledge holds, no commercial TPS has so far been able to provide any option of utilizing LET during the optimization process, or to compute dose distributions weighted by a variable RBE.

To conduct as accurate a clinical evaluation as possible in this circumstance, it is desirable that the deviation of the TPS biological dose that is calculated using a constant RBE from the biological dose computed with a variable RBE, which is herein referred to as a “true” biological dose, is as small as possible.

Thus far, a good number of research works have conducted a comparative study of biological surrogate and LET distributions among different optimization techniques. If the optimization techniques are confined to only those available in commercial TPS, then the recent study by X. Bai et al. shows that robust optimization can reduce both the biological surrogate and the LET at OARs, causing less biological damage to the OARs than PTV‐based plans because the former tends to use lateral fall‐off to spare the OARs rather than the distal edge.^16^ D. Giantsoudi et al. demonstrated a series of Pareto‐optimal IMPT base plans showing substantial LET variations, which leads to potentially considerable differences in RBE‐weighted doses in terms of multicriteria optimization.^17^ These works suggest that different optimization techniques give substantially different biological dose distributions. Conversely, as far as the authors’ knowledge holds, not a single study has explored the correlations between the optimization technique and the deviation of biological doses between the constant and variable RBE. It is therefore seemingly and practically worthwhile to investigate which optimization technique available in commercial TPS gives biological dose distributions closest to those indicated by TPS.

In this research, the authors have looked into the practical perspective of focusing on the PTV‐based and robust plans created using commercial TPS. Biological dose distributions are computed using a variable RBE model, and their deviations from those computed with the constant RBE are evaluated for both the clinical target volume (CTV) and OARs. The influence of the distance between the OARs and CTV on the deviation size is also investigated.

## 2. MATERIALS AND METHODS

### 2.1. Treatment planning

**Fig. 1:**
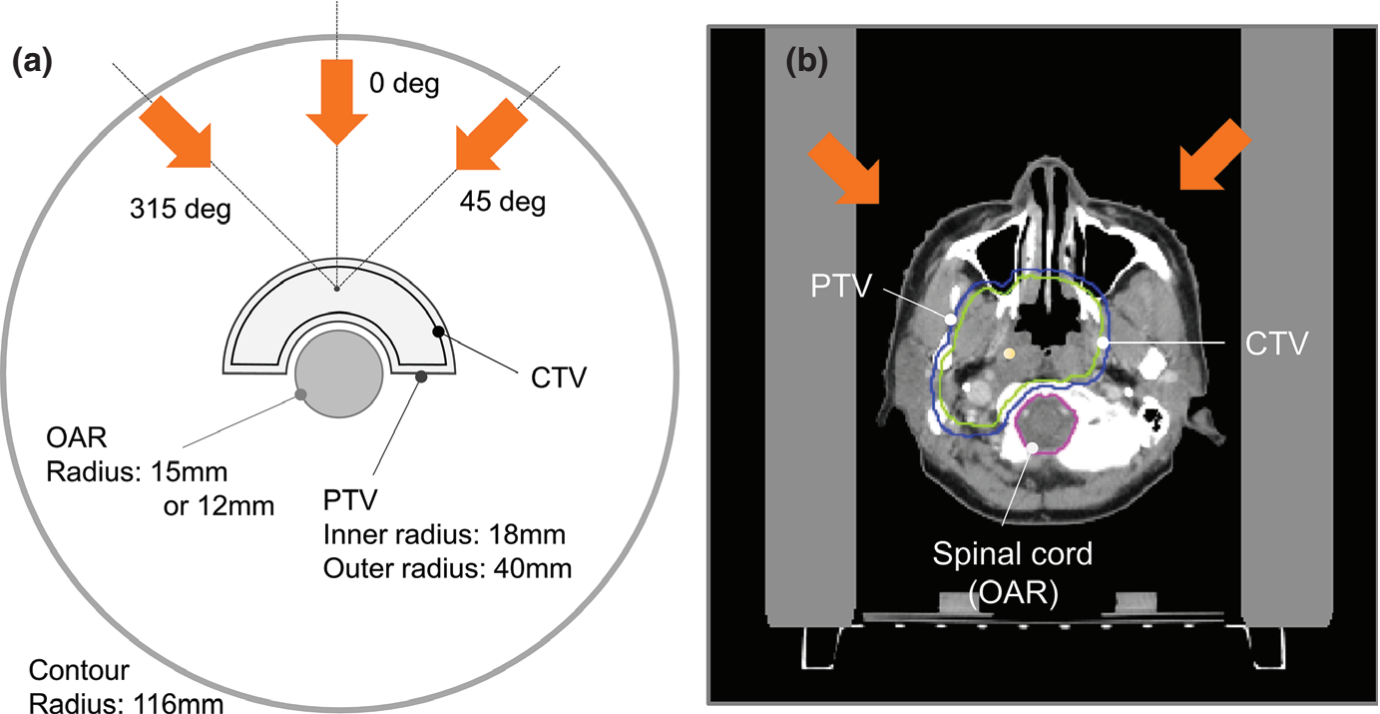
(a) Schematic diagram of the transverse plane of the RTOG benchmark phantom. (b) An example of the transverse plane of the nasopharyngeal case, Case A. Orange arrows indicate the direction of the proton beams. RTOG, Radiation Therapy Oncology Group.

PTV‐based and robust plans were made using VQA (Hitachi Ltd., Tokyo, Japan) for a Radiation Therapy Oncology Group (RTOG) benchmark phantom^2^, ^8^, ^19^ and four nasopharyngeal tumor cases (Fig. 1). In both phantom and patient plans, the PTV was generated by isotropically expanding the CTV by 3 mm.^20^ In the RTOG phantom, different diameters of the OAR (15 and 12 mm) were used to examine the influence of the distance between the OAR and the CTV. The OAR was surrounded by the horseshoe‐shaped PTV with inner and outer radii of 18 and 40 mm, respectively. For the nasopharyngeal cases, the brainstem and spinal cord were regarded as the OARs. The beam angles are also illustrated in Fig. 1.

For the robust plans, a simple voxel‐based worst‐case robust optimization technique was used considering the setup and range uncertainties.^6^, ^18^ The interfractional patient setup uncertainty of 3 mm was incorporated by shifting the isocenter of a patient along the anteroposterior (A‐P), superior–inferior (S–I), and right–left (R–L) directions, while the range uncertainty was set up by scaling the stopping power ratio by −3.5% and 3.5%. The worst‐case dose distribution was represented by the minimum and maximum of the nine doses in each voxel in the CTV, and by the maximum of the nine doses in each voxel in the OARs.

In both PTV‐based and robust plans, the prescription dose, *D*_pres_, was administered to the CTV, such that the *D*_99_ of the CTV > *D*_pres_ (RBE = 1.1). In PTV‐based plans, an additional constraint was applied to the PTV, such that the D_95_ of the PTV > *D*_pres_ (RBE = 1.1). In the robust plans, the minimum *D*_98_ of the CTV among the nine dose distributions, *D*_98,worst_, was made higher than the 95% of the *D*_pres_ (RBE = 1.1).^21^ Spot‐to‐spot intervals were set to 5 mm for both plans.

In the RTOG phantom plan, *D*_pres_ was set to 200 cGy (RBE = 1.1) and the dose constraint was applied to the OAR such that the maximum dose, *D*_max_, of the OAR < 140 cGy (RBE = 1.1). The dose was administered in one fraction. In patient plans, in accordance with the institutional protocol, *D*_pres_ was set to 7140 cGy (RBE = 1.1) and the dose constraints to the spinal cord and the brainstem were set as follows: *D*_max_ of the brainstem < 5400 cGy (RBE = 1.1) and *D*_max_ of the spinal cord < 4600 cGy (RBE = 1.1). The dose was administered in 34 fractions.

### 2.2. Dose and dose‐averaged LET calculations

The variable RBE and the biological dose were obtained by calculating the physical dose and dose‐averaged LET (LET_d_) distributions using analytical methods based on the pencil beam algorithm.^22^, ^23^ In this algorithm, the physical dose was calculated by convolving the fluence with the dose kernel. The dose kernel was represented by a triple Gaussian to include protons that underwent not only multiple Coulomb scattering but also large‐angle scattering due to nuclear reactions.^22^

For the LET_d_ calculation, three‐dimensional LET_d_ distribution of an infinitesimal proton beam in water is defined as the LET kernel. Then, LET_d_ of some point is derived by taking the dose average of all the LET kernels that contribute to that point.^23^ Different LET kernels were created for primary Gaussian dose kernel and second, third Gaussian kernels, respectively. Each LET kernel was assumed to vary only in the depth direction and was constant in the lateral direction. A 2‐mm calculation grid was used in both the dose and LET_d_ calculations.

### 2.3. Biological dose calculation considering LET_d_

The RBE was calculated voxel by voxel using the phenomenological RBE model proposed by McNamara et al.^11^:

(1) $${\text{RBE}}_{i}=-\frac{1}{2d_{i}}{\left(\frac{\alpha }{\beta }\right)}_{x,i}+\frac{1}{d_{i}}\sqrt{\frac{1}{4}{\left(\frac{\alpha }{\beta }\right)}_{x,i}^{2}+\left(0.991+\frac{0.356}{{\left(\alpha /\beta \right)}_{x,i}}L_{d,i}\right){\left(\frac{\alpha }{\beta }\right)}_{x,i}d_{i}+\left(1.101-0.0039\sqrt{{\left(\frac{\alpha }{\beta }\right)}_{x,i}}L_{d,i}\right)d_{i}^{2}},$$

where $d_{i}$, $L_{d,i}$, and ${\left(\alpha /\beta \right)}_{x,i}$ represent the physical dose per fraction, the dose‐averaged LET, and the ${\left(\alpha /\beta \right)}_{x}$ parameter at the i th voxel, respectively. Nasopharyngeal tumor cases were also evaluated using the RBE models proposed by Wilkens et al.^8^ and Wedenberg et al.^10^ These results are shown in the discussion. For the RTOG phantom case, ${\left(\alpha /\beta \right)}_{x}$ parameters for the CTV and OAR were set to 10 and 3 Gy, respectively, while in the nasopharyngeal case, they were set to the values shown in Table 1., 24, 25 The biological dose was calculated by multiplying the RBE [Eq. (1)] and the physical dose in each voxel.

**TABLE 1:** ${\left(\alpha /\beta \right)}_{x}$ parameters for tissues in the nasopharyngeal case.

Tissue	${\left(\alpha /\beta \right)}_{x}$	Reference
CTV (nasopharyngeal tumor)	3 or 12	XK Zheng et al. (2010)^24^
Spinal cord	2.0	D.Giantsoudi et al. (2017)^25^
Brainstem	2.1	D.Giantsoudi et al. (2017)^25^

CTV, clinical target volume

### 2.4. Evaluation

#### 2.4.1. Biological dose analysis

To identify which of the optimization techniques among PTV‐based and robust optimizations gave the “true” biological dose [the biological dose calculated using the variable RBE Eq. (1), $D^{v\text{RBE}}$] closer to that indicated by the TPS ($D^{\text{RBE}=1.1}$), the deviations of $D^{v\text{RBE}}$ from $D^{\text{RBE}=1.1}$ were compared between PTV‐based and robust plans. *D*_99_ and *D*_max_ were used as the evaluation indices for the CTV and OARs, respectively:

(2) $$\Delta D_{99}=\frac{D_{99}^{v\text{RBE}}-D_{99}^{\text{RBE}=1.1}}{D_{\text{pres}}}\times 100,$$

(3) $$\Delta D_{\max}=\frac{D_{\max}^{v\text{RBE}}-D_{\max}^{\text{RBE}=1.1}}{D_{\text{pres}}}\times 100,$$

where the deviations were normalized by the prescribed dose, $D_{\text{pres}}$.

In addition, for the nasopharyngeal case, the authors verified whether the biological dose distribution evaluated with the variable RBE satisfied their institutional criteria of the OARs: $D_{\max}^{v\text{RBE}}$ of the spinal cord < 5000 cGy (RBE) and $D_{\max}^{v\text{RBE}}$ of the brainstem < 6000 cGy (RBE).

#### 2.4.2. Order of CTV‐to‐OAR distance

To investigate whether the distance between the OAR and CTV affected the magnitudes of $\Delta D_{99}$ and $\Delta D_{\max}$, $\Delta D_{99}$ and $\Delta D_{\max}$ were compared between plans with different CTV‐to‐OAR distances. A definition of the order of CTV‐to‐OAR distances is described in the subsequent text.

First, for the RTOG phantom, the closest distance was used as the measure of the distance. Therefore, the OAR was closer to the CTV at an OAR radius of 15 mm. For the nasopharyngeal case, it is not unique to define CTV‐to‐OAR distances because the shapes of the targets and the OARs were more complicated than the RTOG phantom. Consequently, the authors used the overlapped volume histogram (OVH), which is generally exploited to characterize the three‐dimensional spatial relationship between the CTV and the OAR for DVH prediction,^26^, ^27^ to define the order of the CTV‐to‐OAR distance.

The OVH indicated the overlapped volume fraction between the OAR and the tumor when the tumor was expanded at different distances. More specifically, the *k*th element of the OVH for the OAR O, ${\text{OVH}}_{O,k}$, was calculated using the formula

(4) $$\text{OV}H_{O,k}=\frac{\left|\left\{p\in O|d\left(p,\text{CTV}\right)<k\delta \right\}\right|}{\left|O\right|}\times 100\;with\;k=1\ldots \infty ,$$

where $\left|O\right|$ is the volume of the OAR, $d\left(p,\text{CTV}\right)$ is the distance from the position p to the boundary of the CTV, and δ is the finite distance interval, set herein to 3 mm. The numerator represents the subset of the OAR whose distance from the CTV boundary is less than kδ. In this study, OVH was evaluated at *k* = 3 and greater value of ${\text{OVH}}_{O,3}$ was regarded as the geometry with closer CTV‐to‐OAR distance.

## 3. RESULTS

### 3.1. RTOG benchmark phantom

Table 2 shows the dosimetric parameters, $\Delta D_{99}$, and $\Delta D_{\max}$, of the PTV‐based and robust plans. When RBE = 1.1, all dosimetric parameters showed slight differences among the optimization techniques. When evaluated with a variable RBE, the values of $D_{99}$ decreased from those evaluated with RBE = 1.1, but the size of $\Delta D_{99}$ was similar between optimization techniques in both RTOG phantoms (OAR radii of 15 and 12 mm). On the contrary, the values of $D_{\max}$ increased from those evaluated with RBE = 1.1 in all optimization techniques and phantoms. For the RTOG phantom with the smaller CTV‐to‐OAR distance (OAR radius of 15 mm), the magnitude of $\Delta D_{\max}$ was 14.5% and 6.5% for PTV‐based and robust plans, respectively, which indicates that the latter yielded a biological dose distribution much closer to that displayed by the TPS. For the RTOG phantom with the larger CTV‐to‐OAR distance (OAR radius of 12 mm), the magnitude of $\Delta D_{\max}$ was 11.5% and 10.5% for PTV‐based and robust plans, respectively. Indeed, the closeness of the “true” biological dose to that displayed by the TPS became similar among the optimization techniques with an increasing CTV‐to‐OAR distance.

**TABLE 2:** Comparison of the dosimetric parameters in the RTOG phantom plans with RBE = 1.1 (equal to the treatment plan) and a variable RBE.

OAR radius	Tissue	Dosimetric parameter	PTV‐based opt. [cGy(RBE)]			Robust opt. [cGy(RBE)]		
			RBE = 1.1	Variable RBE	$\Delta D_{99},\Delta D_{\max}$[%]	RBE = 1.1	Variable RBE	$\Delta D_{99},\Delta D_{\max}$[%]
15 mm	CTV	*D*_99_	209.5	207.5	−1	203.5	202.5	−0.5
		*D*_98,worst_	–	–		190.5	–	
	PTV	*D*_95_	203.5	–		–	–	
	OAR	*D*_max_	131.5	160.5	14.5	135.5	148.4	6.5
12 mm	CTV	*D*_99_	202.5	196.5	−3	203.5	197.5	−3
		*D*_98,worst_	–	–		200.5	–	
	PTV	*D*_95_	201.5	–		–	–	
	OAR	*D*_max_	133.5	156.5	11.5	128.5	149.4	10.5

CTV, clinical target volume; OARs, organs at risk; PTV, planning target volume; RBE, relative biological effectiveness; RTOG, Radiation Therapy Oncology Group.

**Fig. 2:**
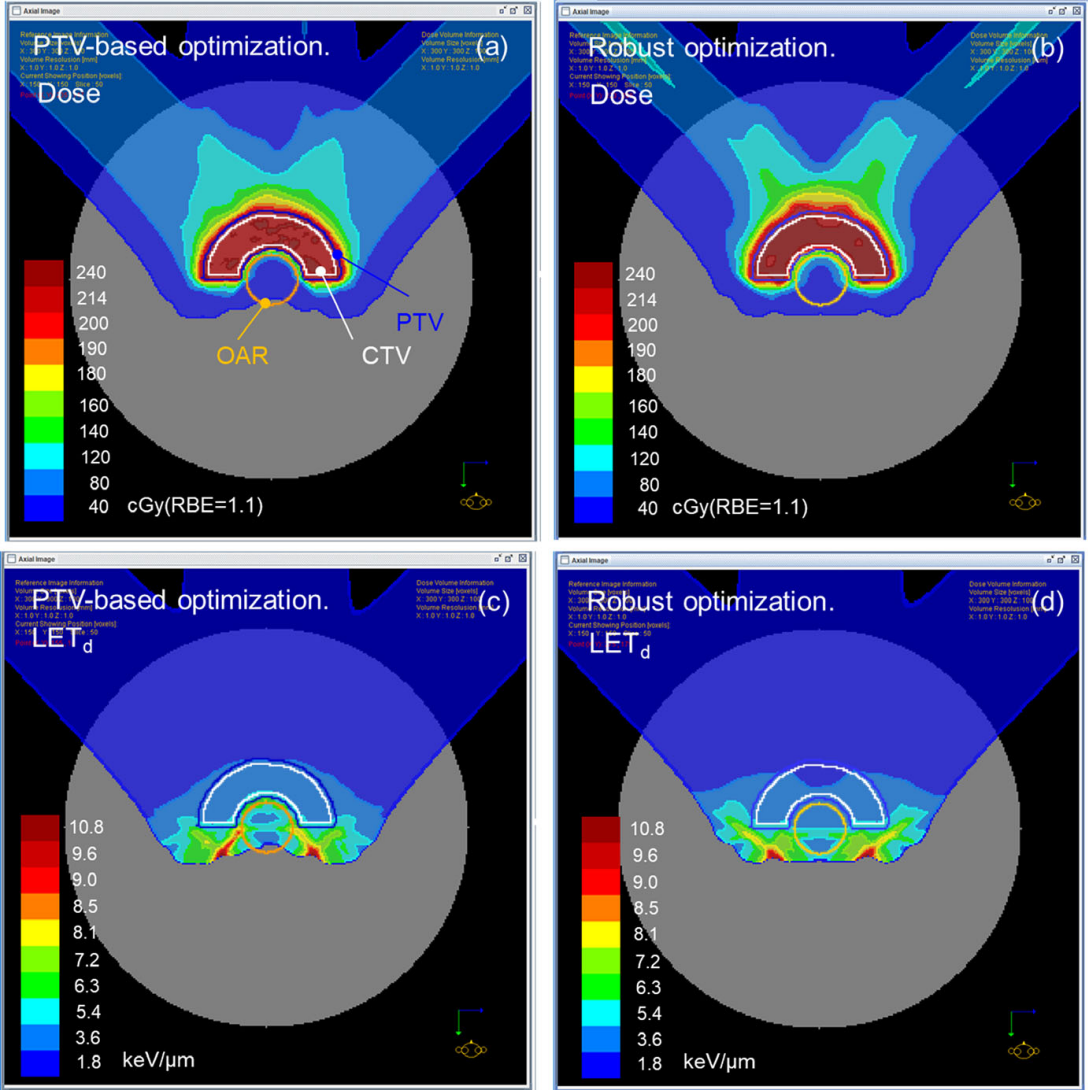
(a, b) Biological dose distributions displayed by the TPS (RBE = 1.1) for PTV‐based and robust plans created for the RTOG phantom with an OAR radius of 15 mm. (c, d) LET_d_ distributions corresponding to (a) and (b), respectively. LET, linear energy transfer; OARs, organs at risk; PTV, planning target volume; RBE, relative biological effectiveness; RTOG, Radiation Therapy Oncology Group; TPS, treatment planning systems.

Figure 2 shows the biological dose distributions displayed by the TPS (RBE = 1.1) and the corresponding LET_d_ distributions with the RTOG phantom with the OAR radius of 15 mm. Comparing the LET_d_ distributions in the two plans, it could be observed that the PTV‐based plan possessed a higher LET_d_ region at the vicinity of the OAR than the robust plans, which resulted from the PTV‐based optimization trying to reduce the OAR dose using the distal edge where the LET_d_ was enhanced rapidly. This is in contrast to the robust plan where the OAR tended to be spared using lateral penumbra.

### 3.2. Nasopharyngeal tumor cases

Table 3 shows the dosimetric parameters, $\Delta D_{99}$, and $\Delta D_{\max}$, of the PTV‐based and robust plans from cases A to D of the nasopharyngeal tumors. In the robust plans, the $D_{\max}$ evaluated with the variable RBE satisfied the authors’ institutional criteria described in Section 2.4.1, whereas in the PTV‐based plans, two of four plans did not fulfill the criteria.

**TABLE 3:** Comparison of the dosimetric parameters for nasopharyngeal tumor cases with RBE = 1.1 (equal to the treatment plan) and the variable RBE.

Case	Tissue	Dosimetric parameter	PTV‐based opt. (cGy [RBE])			Robust opt. (cGy [RBE])		
			RBE = 1.1	Variable RBE	$\Delta D_{99},\Delta D_{\max}$[%]	RBE = 1.1	Variable RBE	$\Delta D_{99},\Delta D_{\max}$[%]
CaseA	CTV	*D*_99_	7220	7534/6987	4.4/−3.3	7223	7510/6980	4.0/−3.4
		*D*_98,worst_	–	–		7040	–	
	PTV	*D*_95_	7236	–		–	–	
	Brainstem	*D*_max_	3893	4947	15.2	4029	4675	9.0
	Spinal cord	*D*_max_	4199	5593	19.5	4199	4947	10.5
CaseB	CTV	*D*_99_	7215	7531/6994	4.4/−3.1	7211	7585/7052	5.2/−2.2
		*D*_98,worst_	–	–		7042	–	
	PTV	*D*_95_	7232	–		–	–	
	Brainstem	*D*_max_	4505	5967	20.5	4573	5355	11.4
	Spinal cord	*D*_max_	2907	3893	13.8	2805	3417	8.6
CaseC	CTV	*D*_99_	7198	7504/6960	4.3/−3.3	7221	7589/7028	5.2/−2.7
		*D*_98,worst_	–	–		7039	‐	
	PTV	*D*_95_	7215	–		–	–	
	Brainstem	*D*_max_	4845	6001	16.2	4845	5797	13.3
	Spinal cord	*D*_max_	3553	4743	16.7	3281	4233	13.3
CaseD	CTV	*D*_99_	7225	7531/7055	4.3/−2.4	7225	7463/6953	3.3/−3.8
		*D*_98,worst_	–	–		7157	–	
	PTV	*D*_95_	7191	–	16.7	–	–	13.3
	Brainstem	*D*_max_	3859	5015	16.2	3689	4675	13.8
	Spinal cord	*D*_max_	3077	4199	15.7	3043	3995	13.3

As for the dosimetric parameters for the CTV calculated with the variable RBE, the results of ${\left(\alpha /\beta \right)}_{x}=3$ and ${\left(\alpha /\beta \right)}_{x}=12$ are shown in the left and right sides in the same cell, respectively. Values of the dosimetric parameters are rounded to the nearest 0.1 cGy.

CTV, clinical target volume; PTV, planning target volume; RBE, relative biological effectiveness.

**Fig. 3:**
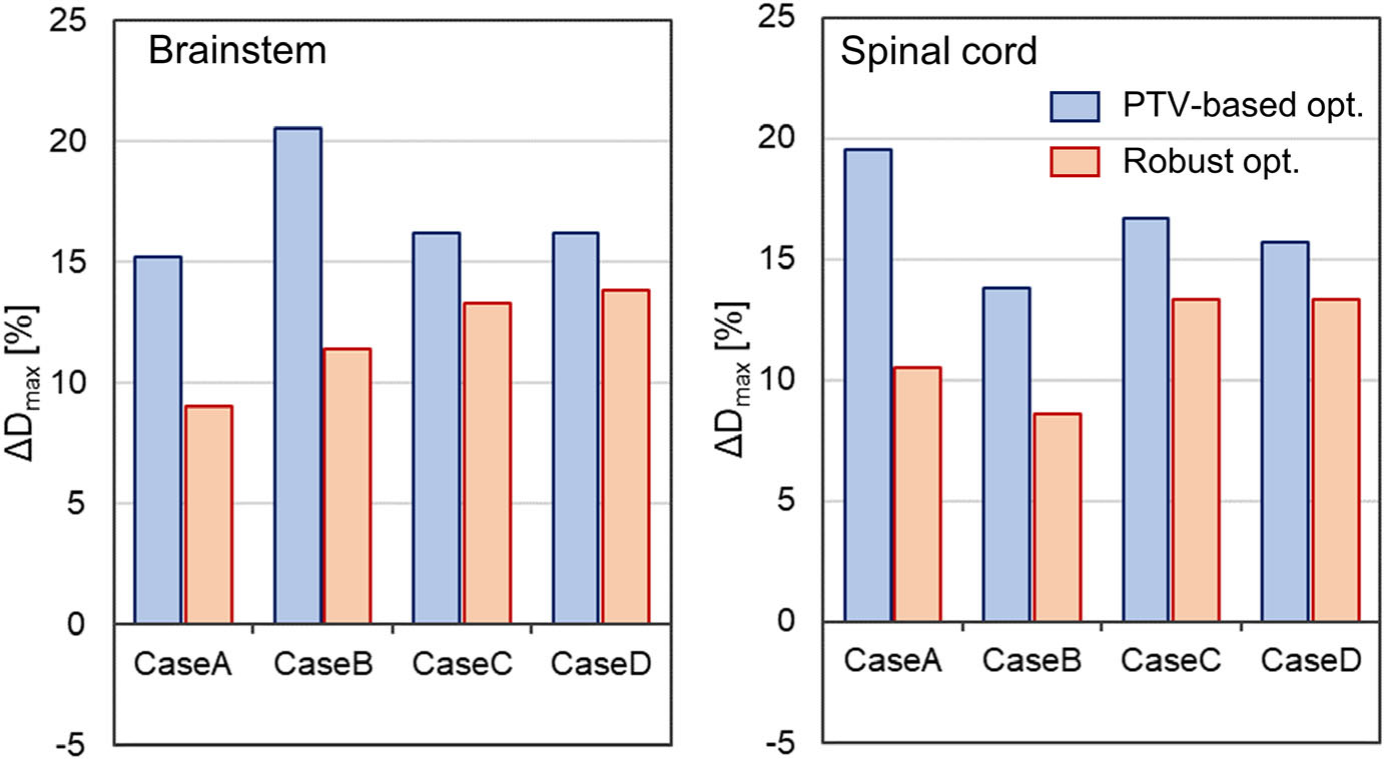
Comparison of the $\Delta D_{\max}$ for the OARs between PTV‐based (blue) and robust (red) plans in the nasopharyngeal case: (a) brainstem and (b) spinal cord. OARs, organs at risk; PTV, planning target volume.

Figure 3(a) and 3(b) presents the comparison of $\Delta D_{\max}$ for the brainstem and the spinal cord in the four nasopharyngeal cases. In all cases, PTV‐based plans resulted in larger $\Delta D_{\max}$ values than the robust plans. The maximum $\Delta D_{\max}$ value among the four cases was + 20.5% for the brainstem and + 19.5% for the spinal cord in the PTV‐based plans, whereas it was + 13.8% for the brainstem and + 13.3% for the spinal cord in the robust plans.

**Fig. 4:**
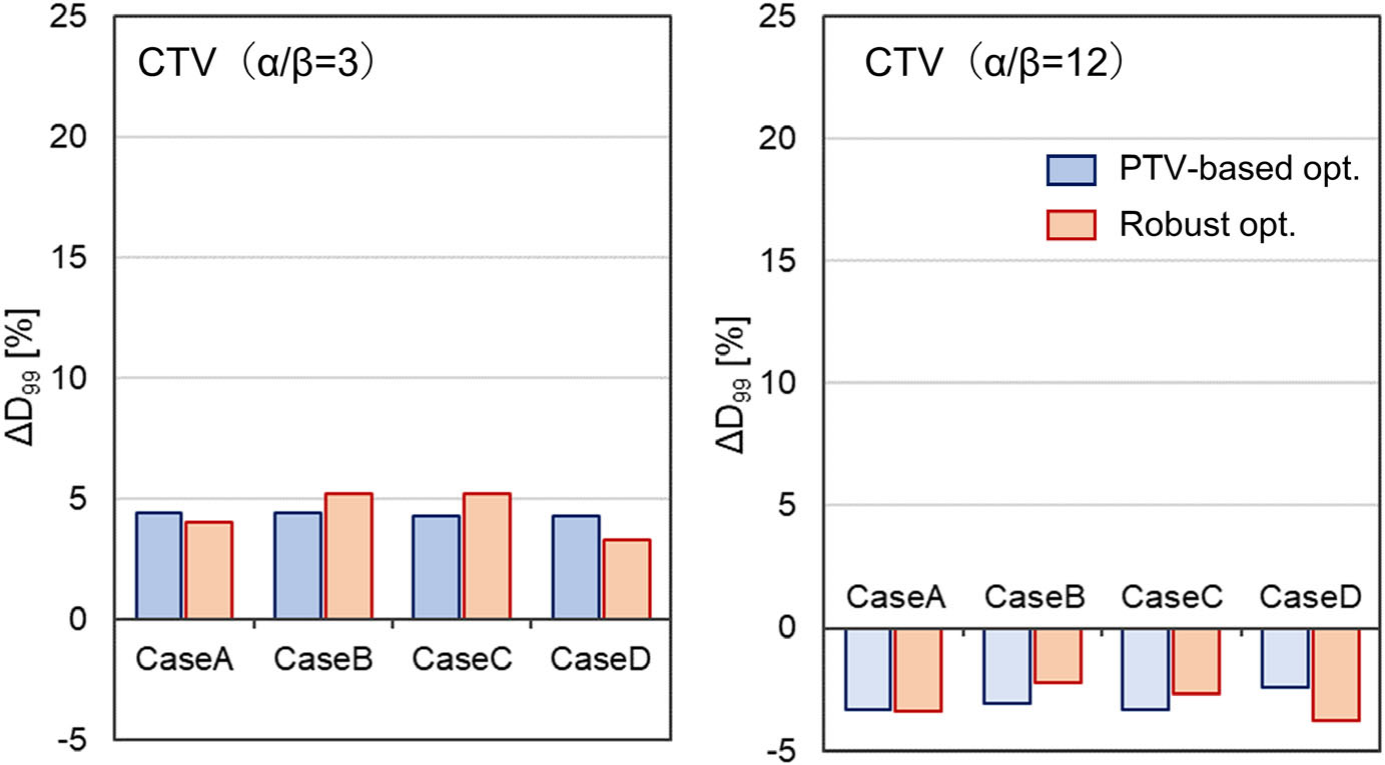
Comparison of the $\Delta D_{99}$ for the CTV between PTV‐based (blue) and robust (red) plans in the nasopharyngeal case: (a)${\left(\alpha /\beta \right)}_{x}=3$ and (b) ${\left(\alpha /\beta \right)}_{x}=12$. CTV, clinical target volume; PTV, planning target volume.

Figure 4(a) and 4(b) shows the comparison of the $\Delta D_{99}$ for the CTV in the case of ${\left(\alpha /\beta \right)}_{x}=3$ and ${\left(\alpha /\beta \right)}_{x}=12$, respectively. The difference in the $\Delta D_{99}$ between the optimization techniques did not have any meaningful correlation with the value of ${\left(\alpha /\beta \right)}_{x}$. The maximum value of the $\Delta D_{99}$ (${\left(\alpha /\beta \right)}_{x}=3$) was + 4.4% in the PTV‐based plan and + 5.2% in the robust plan, whereas its minimum value (${\left(\alpha /\beta \right)}_{x}=12$) was −3.3% in the PTV‐based plan and − 3.8% in the robust plan.

Table 4 shows the ${\text{OVH}}_{O,3}$ of the brainstem and the spinal cord for all four cases. As described in Section 2.4.2, the greater value of ${\text{OVH}}_{O,3}$ was regarded as the geometry with closer CTV‐to‐OAR distance. For the brainstem, the order of ${\text{OVH}}_{O,3}$ was case B > case A > case C > case D. This was in line with the descending order in the difference of $\Delta D_{\max}$ between optimization techniques; the differences ranged from 9.1% (case B) to 2.4% (case D). For the spinal cord, the order of ${\text{OVH}}_{O,3}$ was case A > case B > case C > case D. This was also in line with the descending order in the difference of $\Delta D_{\max}$ between optimization techniques; the differences ranged from 9.0% (case A) to 2.4% (case D). These findings indicate that the difference in $\Delta D_{\max}$ between the optimization techniques increases as the CTV‐to‐OAR distance decreases, as was observed in the cases of the RTOG phantom.

**TABLE 4:** The values of ${\text{OVH}}_{O,3}$ [%] of the brainstem and the spinal cord for cases A, B, C, and D. The value of ${\text{OVH}}_{O,3}$ corresponds to the subset of the OAR whose distance from the CTV boundary is less than 9 mm.

	case A	case B	case C	case D
Brainstem	7.7	13.1	6.8	0
Spinal cord	1.4	0.3	0.1	0

CTV, clinical target volume; OARs, organs at risk; OVH, overlapped volume histogram.

## 4. DISCUSSION

In this study, the deviations of the biological doses indicated by the TPS from those computed with the variable RBE model were compared between PTV‐based and robust plans, which were created using a commercial TPS. For the CTV, the difference in the magnitude of $\Delta D_{99}$ between the optimization techniques was negligible in both the RTOG phantom and nasopharyngeal cases, regardless of the values of the parameter ${\left(\alpha /\beta \right)}_{x}$. For the OARs, the $\Delta D_{\max}$ for the robust plans was much smaller than that for the PTV‐based plans (see Table 2 and Fig. 3), which indicates that the biological dose derived by the TPS is closer to the “true” biological dose, and thus is more reliable in robust plans than in PTV‐based plans. A similar trend was observed in the biological surrogate according to the study conducted by X. Bai et al.^16^ With this, the authors carried out further analysis of the correlation between the difference of $\Delta D_{\max}$ among the optimization techniques and the CTV‐to‐OAR distance, which showed a large value when the OAR was closer to the CTV.

**Fig. 5:**
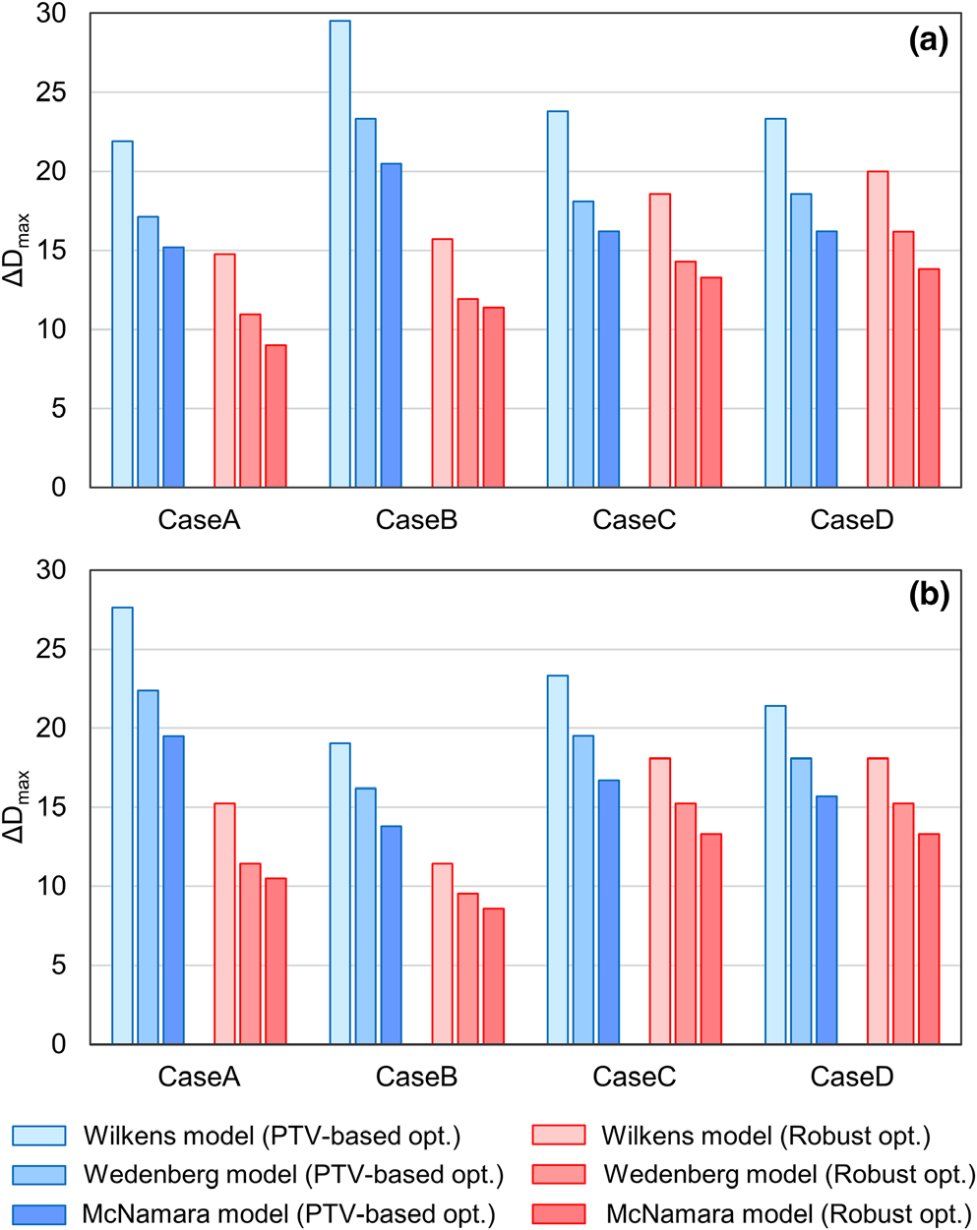
Comparison of the $\Delta D_{\max}$ for the OARs between PTV‐based (blue) and robust plans (red) in the nasopharyngeal case: (a) Brainstem, (b) Spinal cord. From left to right in each set of bars, the results calculated with RBE models proposed by Wilkens et al., Wedenberg et al., and McNamara et al. are shown. OARs, organs at risk; PTV, planning target volume; RBE, relative biological effectiveness.

**Fig. 6:**
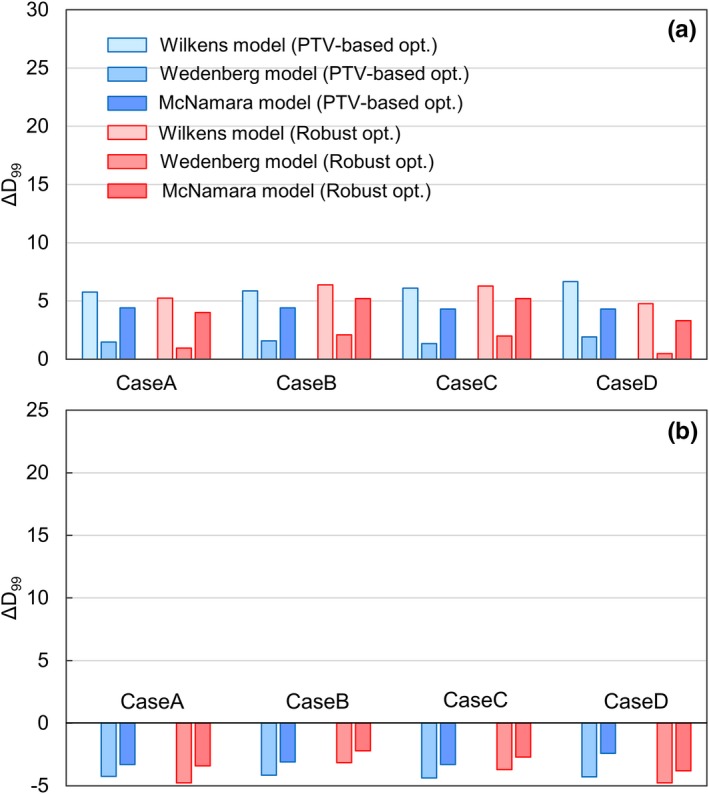
Comparison of the $\Delta D_{99}$ for the CTV between PTV‐based (blue) and robust plans (red) in the nasopharyngeal case: (a)${\left(\alpha /\beta \right)}_{x}=3$, (b) ${\left(\alpha /\beta \right)}_{x}=12$. From left to right in each set of bars, the results calculated with RBE models proposed by Wilkens et al., Wedenberg et al., and McNamara et al. are shown. CTV, clinical target volume; PTV, planning target volume; RBE, relative biological effectiveness.

Although the results shown were from the variable RBE model developed by McNamara et al, other RBE models such as Wilkens et al.^8^ and Wedenberg et al.^10^ were found to show the same tendencies. Figure 5(a) and 5(b) presents the comparison of $\Delta D_{\max}$ for the brainstem and the spinal cord, respectively, in the four nasopharyngeal cases, evaluated using Wilkens et al., Wedenberg et al., and McNamara et al. In all RBE models, PTV‐based plans resulted in larger $\Delta D_{\max}$ values than the robust plans for all cases. In addition, the difference of $\Delta D_{\max}$ between the optimization techniques was larger when the OAR was closer to the CTV. Among the RBE models, Wilkens et al. showed largest $\Delta D_{\max}$, followed by Wedenberg et al. and McNamara et al. This is the same trend observed in RBE for low ${\left(\alpha /\beta \right)}_{x}$ in the high LET region reported in the literature.^29^ Figure 6(a) and 6(b) shows the comparison of the $\Delta D_{99}$ for the CTV in the case of ${\left(\alpha /\beta \right)}_{x}=3$ and ${\left(\alpha /\beta \right)}_{x}=12$, respectively. Note that the Wilkens RBE model does not have a ${\left(\alpha /\beta \right)}_{x}$ dependency,^8^ and thus was not included in both figures. In contrast to $\Delta D_{\max}$, the difference in the $\Delta D_{99}$ between optimization techniques was negligible in all RBE models for both ${\left(\alpha /\beta \right)}_{x}=3$ and 12 Gy. In the case of ${\left(\alpha /\beta \right)}_{x}=3$, the $\Delta D_{99}$ calculated with the Wedenberg RBE model was smaller than McNamara RBE model. This probably resulted from the fact that in the case of low ${\left(\alpha /\beta \right)}_{x}$, the Wedenberg model gives a smaller RBE than the McNamara model in uniform dose regions where LET is small.^11^, ^29^

The above description suggests the potential that applying robust optimization may reduce $\Delta D_{\max}$, especially when the OAR is close to the tumor. However, it should be noted that even with robust optimization, in certain cases, the $\Delta D_{\max}$ of the OAR was still large (more than 13% in cases C and D, as depicted in Fig. 3). Adding explicit terms in consideration of the LET distribution to the objective function of IMPT enables the control of the LET distribution, for example, by suppressing LET to OARs and/or concentrating LET on tumors. In fact, different versions of LET optimization techniques in this regard have been proposed by several groups,^14^, ^15^, ^28^ and recently, techniques have been developed to specifically incorporate the optimization of the LET_d_ distribution into robust optimization.^13^, ^15^ Using these techniques, it should become possible to further reduce the deviation not only for the OARs close to the tumor but also for the OARs distant from the tumor.

As described above, robust optimization has an advantage that it is capable of handling not only the physical uncertainty against setup and range errors but also (though not intentionally) the uncertainty against the biological dose. Though we have shown that the maximum biological dose in OAR is smaller in robust plans, the OAR volume receiving a low dose is larger than PTV‐based plans, as shown in Fig. 2. This is because the robust plans often use the lateral penumbra to avoid the OAR instead of distal fall‐off. Therefore, it should be decided which optimization to be used in clinics within these trade‐offs.

Finally, as the scope of this study focused on the comparison between the RTOG phantoms and nasopharyngeal cases with only the brainstem and the spinal cord examined as the OARs, the authors believe that the findings herein could be established in more general settings in a future study that involves the use of different treatment sites and OARs.

## 5. CONCLUSION

Under the circumstance that the current commercial TPS indicates only the biological dose evaluated with a constant RBE, it is of practical importance that such biological doses derived by the TPS should be as close to the “true” biological dose (the biological dose calculated with variable RBE) as possible. The result of the comparison between the PTV‐based and robust plans of the RTOG phantom and nasopharyngeal tumor cases indicated that the deviations of $D_{\max}^{v\text{RBE}}$ from $D_{\max}^{\text{RBE}=1.1}$ of OARs tend to be smaller in robust plans as compared to PTV‐based plans. In addition, the deviation becomes larger as the OAR is located closer to the CTV. Similar tendencies were observed in three different RBE models. Therefore, robust optimization was found to be more favorable than PTV‐based optimization in that the results presented by the TPS were closer to the "true" values, and thus clinical evaluation based on these results will be more reliable when employing robust optimization.

## CONFLICT OF INTERESTS

We disclose Shusuke Hirayama and Takaaki Fujii are paid from Hitachi, Ltd., Tokyo, Japan.
